# Functional classification of grasp strategies used by hemiplegic patients

**DOI:** 10.1371/journal.pone.0187608

**Published:** 2017-11-10

**Authors:** Alicia García Álvarez, Agnès Roby-Brami, Johanna Robertson, Nicolas Roche

**Affiliations:** 1 Department of Physical Medicine and Rehabilitation, CHU Raymond Poincare - APHP, Garches, France; 2 Institut des Systèmes Intelligents et de Robotique, CNRS, University Pierre et Marie Curie - Sorbonne Universities, Paris, France; 3 Department of Physical Medicine and Rehabilitation, CHU Nantes, Nantes, France; 4 Department of Physiology, University of Versailles Saint-Quentin-en-Yvelines U1179, Garches, France; University of Ottawa, CANADA

## Abstract

This study aimed to identify and qualify grasp-types used by patients with stroke and determine the clinical parameters that could explain the use of each grasp. Thirty-eight patients with chronic stroke-related hemiparesis and a range of motor and functional capacities (17 females and 21 males, aged 25–78), and 10 healthy subjects were included. Four objects were used (tissue packet, teaspoon, bottle and tennis ball). Participants were instructed to “grasp the object as if you are going to use it”. Three trials were video-recorded for each object. A total of 456 grasps were analysed and rated using a custom-designed Functional Grasp Scale. Eight grasp-types were identified from the analysis: healthy subjects used Multi-pulpar, Pluri-digital, Lateral-pinch and Palmar grasps (Standard Grasps). Patients used the same grasps with in addition Digito-palmar, Raking, Ulnar and Interdigital grasps (Alternative Grasps). Only patients with a moderate or relatively good functional ability used Standard grasps. The correlation and regression analyses showed this was conditioned by sufficient finger and elbow extensor strength (Pluri-digital grasp); thumb extensor and wrist flexor strength (Lateral pinch) or in forearm supinator strength (Palmar grasp). By contrast, the patients who had severe impairment used Alternative grasps that did not involve the thumb. These strategies likely compensate specific impairments. Regression and correlation analyses suggested that weakness had a greater influence over grasp strategy than spasticity. This would imply that treatment should focus on improving hand strength and control although reducing spasticity may be useful in some cases.

## Introduction

Stroke is the leading cause of morbidity and the third cause of mortality (50000 deaths per year) in industrialised countries. The annual incidence is around 300 per 100 000 inhabitants, equivalent to 125 000 new cases each year in France. Around half of survivors are left with some disability [1] as a result of multiple impairments that often involve a loss of strength, stereotyped movements and changes in muscle tone. These impairments limit the capacity to carry out activities of daily living (ADL). The upper limb is particularly affected with 30% of patients left with a ‘plegic’ upper limb. These patients are unable to move the limb and thus are partially or totally dependent for ADL such as dressing, washing and feeding. Another 40% of patients have some proximal recovery and are able to move their shoulder and sometimes the elbow. They can use their upper limb for certain ADL such as carrying a bag or stabilizing paper for writing. The final 30% recover the possibility to grasp. However, because of altered motor control, both reaching and grasping are often impaired. [2]

The normal kinematics of reaching and grasping movements were demonstrated by the pioneering work of Jeannerod [3]. Jeannerod showed that the two components, reaching and grasping, evolve in parallel as a function of the position, orientation and size of the object, with a smooth velocity profile. The hand and fingers are pre-shaped during the reach according to the size [4], shape and the function of the object [5,6,7]. The configuration of the hand to grasp an object or a tool is intimately related to the nature of the activity to be carried out [8,9,10,11,12].

Following stroke, these kinematics are altered. The velocity profile of the paretic upper limb is slow and segmented [13,14] and the trajectory of the hand is more curved and less fluid [15,16]. This is due to muscle co-contractions that reduce selective control, as well as muscle weakness and abnormal muscle excitability [13]. The capacity to open the hand during the reach is also reduced [17] as a result of a decrease in activation of the finger extensors [18,19,2] and a loss of coordination between the flexor and extensor muscles [20]. The pre-shaping of the hand that normally occurs prior to the grasp is also delayed [21,22].

In healthy subjects, there is a strong coupling between grasping and lifting forces during the displacement of an object [23,24]. Stroke results in a difficulty to adapt the level of force required to grasp or manipulate an object as well as to rapidly modify the forces applied [24]. The loss of dexterity following stroke results in a reduction of functional capacity [24,25,26]. Both fine motor control of grasping (precision grip between the thumb and index finger) and gross motor control of grasping (power grip between the thumb and the other fingers) are affected.

The great flexibility of the human hand to grasp and manipulate objects [27] has been mainly studied using qualitative methods. Napier [28] proposed a dichotomous classification of grasping: “precision grip” and “power grasp”, which are employed depending on the requirement of the task. Later Iberall et al [29] added an intermediate “key grip” and proposed a different description of grasping based on the direction of the forces applied by the fingers relative to the three axes of the palm. They also defined virtual fingers as a unit of several fingers (or palm) working together in opposition to form the grasp. The taxonomy of grasping was further developed by subdivision of the three classes (precision, power and intermediate) as a function of the hand configuration [30,31].

Despite the importance of hand function in daily life, the strategies used by patients with stroke to grasp objects have been little studied. Bensmail et al [32] studied grasping strategies in 15 patients with hemiparesis, based on video analysis. They used four objects of different shapes and weights (a ball, cone, cylinder and square) and found that the patients used 7 different grasp strategies (raking, palmar, inter-digital, intrinsic, multi-pulpar, ulnar and pulpo-lateral) to grasp the 4 objects. However the reasons for the strategies used by a given patient were not explored. The level of recovery differed between the patients thus it could be hypothesised the grasp-type was determined by different impairments, such decreased range passive of motion, muscle weakness, spasticity or loss of sensation.

The aim of the present study was to qualify grasp-types used by stroke patients and to identify the clinical parameters that could explain the use of each grasp. The long term objective was to guide treatment, in particular rehabilitation and botulinum toxin injections.

## Method

### Participants

Outpatients followed in the Spasticity Department of Raymond-Poincaré Hospital, Garches, France were recruited. Inclusion criteria were: single unilateral stroke of non-traumatic origin at least one year previously and last botulinum toxin injection at least 4 months before inclusion (for patients undergoing this treatment). Patients with cognitive impairments, shoulder pain or other neurological or orthopaedic disorders were excluded. Fifty potentially eligible patients were assessed between 1^st^ October 2013 and 31^st^ April 2014. Of these, 38 were able to grasp an object and consented to participate and were therefore included (Table 1). The study was performed in accordance with the ethical codes of the World Medical Association (Declaration of Helsinki) and was approved by our local Ethics Committee (CPP Ile de France XI– 78105 Saint Germaine en Laye, number 12071). All patients signed an informed consent form. Ten healthy subjects with no history of neurological disease, orthopaedic surgery to their dominant upper limb or current pain in the dominant upper limb were also included to constitute the control group (Table 1).

**Table 1:** Participant characteristics.

Characteristic	Stroke Group (n = 38)	Control Group (n = 10)
Gender (male/female)	21/17	7/10
Age, years (SD)	54 (15)	32 (6)
Handedness (right/left)	35/3	9/1
Time since stroke, months (IQ)	102 (101)	N/A
Side of paresis (right/left)	16/22	N/A
Spasticity in the affected hand (yes/no)	30/8	N/A
FMA-UE (0–66)(IQ)	38.5* (14)	N/A
ARAT (0–57)(IQ)	25* (17)	N/A
Upper limb sensation (impaired/not impaired)	19/19	N/A
Upper limb proprioception (impaired/not impaired)	15/23	N/A

IQ: Interquartile, FMA-UE: Fugl-Meyer Assessment-Upper Extremity, ARAT: Action Research Arm Test.

N/A: not applicable,

*median.

### Clinical evaluation

Each patient underwent a clinical evaluation that included:

- Passive joint Range of Motion (ROM) using a manual goniometer: shoulder abduction, flexion/extension and internal/external rotation; elbow: flexion/extension; forearm pronation/supination and wrist and fingers: flexion/extension
- Strength graded according to the *Medical Research Council Scale* (MRC): shoulder abductors and internal/external rotators, elbow flexors /extensors, forearm supinator/pronators, wrist flexors/extensors, finger flexors/extensors and thumb flexors/extensors.
- Spasticity graded according to the *Modified Ashworth Scale* (MAS): shoulder adductors and internal rotators, elbow flexors/extensors, forearm pronator/supinators, wrist flexors-extensors, finger flexors and thumb flexor, adductor and opponent muscles.
- Proprioception of the proximal and distal parts of the arm: shoulder, arm, forearm and hand. Sensation of the forearm and hand. Both were rated from 1 to 3: 1 = anaesthesia, 2 = hypoesthesia and 3 = normal sensation or proprioception. For proprioception the test of position sense was used: the patient’s eyes were closed and the investigator placed the hemiparetic arm in a determined position that the patient was asked to reproduce with the non-hemiparetic arm. Light touch was tested by stroking the patient’s arm and hand with examiner fingers, the patient was asked to compare the sensation with the non-affected arm.
- Function: Action Research Arm Test (ARAT) [33]: this test is commonly used to assess upper limb function following stroke. It assesses the patient’s ability to grasp and displace objects of different sizes, weights and shapes. It consists of 19 items divided into 4 sub-tests (grasp, grip, pinch, and gross arm movement). Performance on each item is rated on a 4-point ordinal scale: 3: performs test normally, 2: completes test, but takes abnormally long or has great difficulty, 1: performs test partially and 0: can perform no part of test. The total score is out of 57. The test has been shown to be reliable and valid for use in patients with stroke [34].
- Impairment: Fugl Meyer Assessment for Upper Extremity (FMA-UE) [35,36]: this test is widely used to assess motor recovery following stroke. Particularly, it evaluates the capacity to move limb segments independently from other segments. The upper-extremity motor section of this test was used in this study. Items are scored on a 3-point ordinal scale: 0 = cannot perform, 1 = performs partially, 2 = performs fully. The maximal score for this section is 66 points. The FMA test is valid and reliable when administered by trained therapist [34]. More details about ARAT and FMA-UE scales are presented in S1 Text.

### Protocol

Each patient participated in a single visit during which 4 grasping tasks were carried out in a randomised order with the affected hand and were video-recorded. The clinical examination was carried out on the same day.

The healthy subjects carried out the same grasping tasks with their dominant hand, which were also video-recorded.

#### Experimental set up

**Fig 1:**
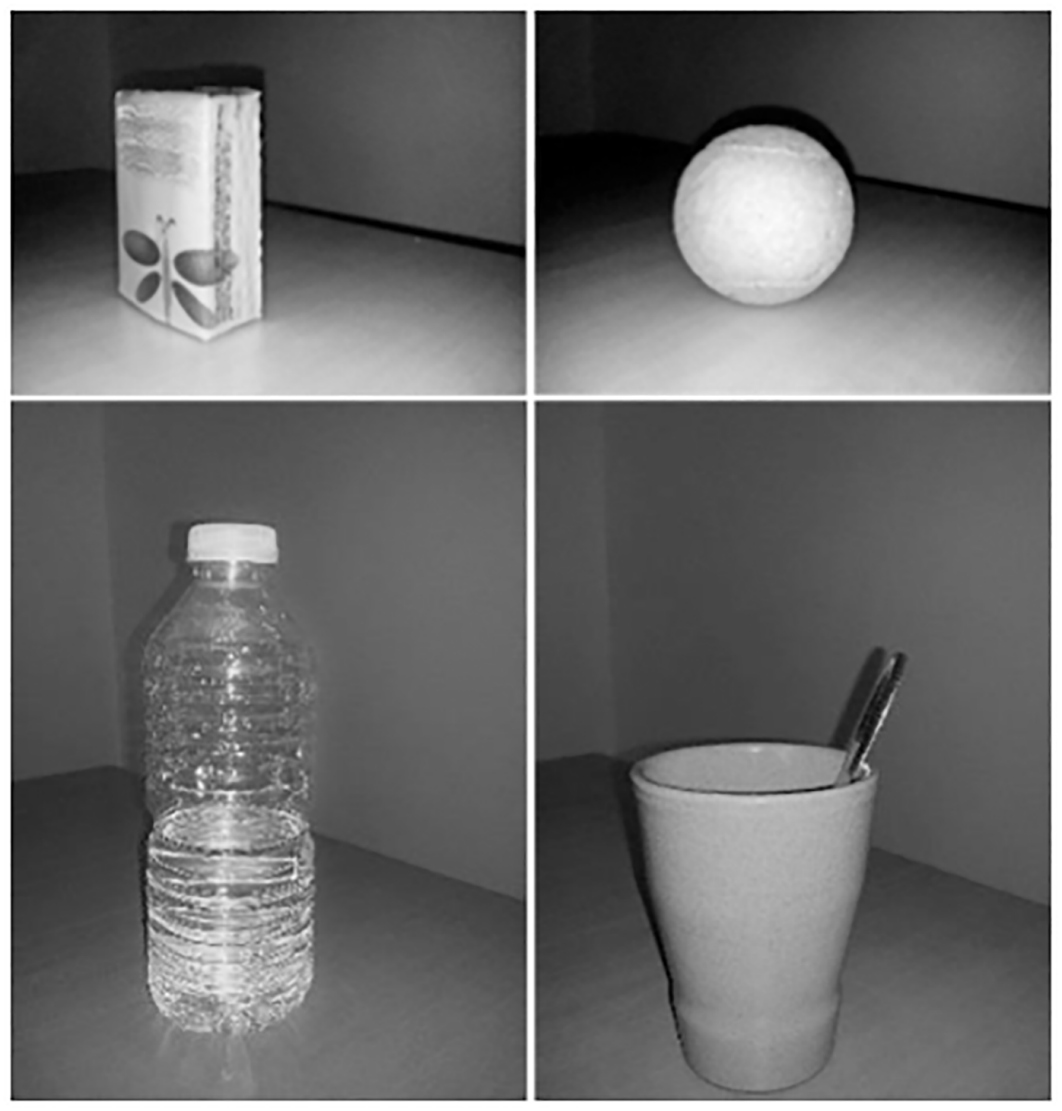
Objects used to assess grasping. Half-filled water bottle (height 22 cm, upper diameter 3 cm and lower diameter 6cm, weight 260 grams); Teaspoon (length 14 cm, thickness 1 mm, weight 90 grams), Packet of paper tissues (height 7.5 cm, length 5 cm, thickness 2.5 cm, weight 20 grams) and Tennis ball (diameter 6 cm, weight 58 grams).

Analysis of grasping: The four objects are shown in Fig 1: a half-filled water bottle, a teaspoon, a packet of paper tissues and a tennis ball. These objects were chosen because 1) they are commonly used in daily life and 2) they provide a variety of shapes, sizes and weights, affording different grasp configurations.

The participants sat on a chair (48cm high) in front of a table (70 cm high). The length of the paretic arm was measured (from the acromion process to the wrist joint line) and the object was placed on the table in the sagittal plane at 30% of arm’s length. This distance was chosen to ensure that the objects could be easily grasped by patients with limited elbow extension without using compensatory trunk movements [37].

**Fig 2:**
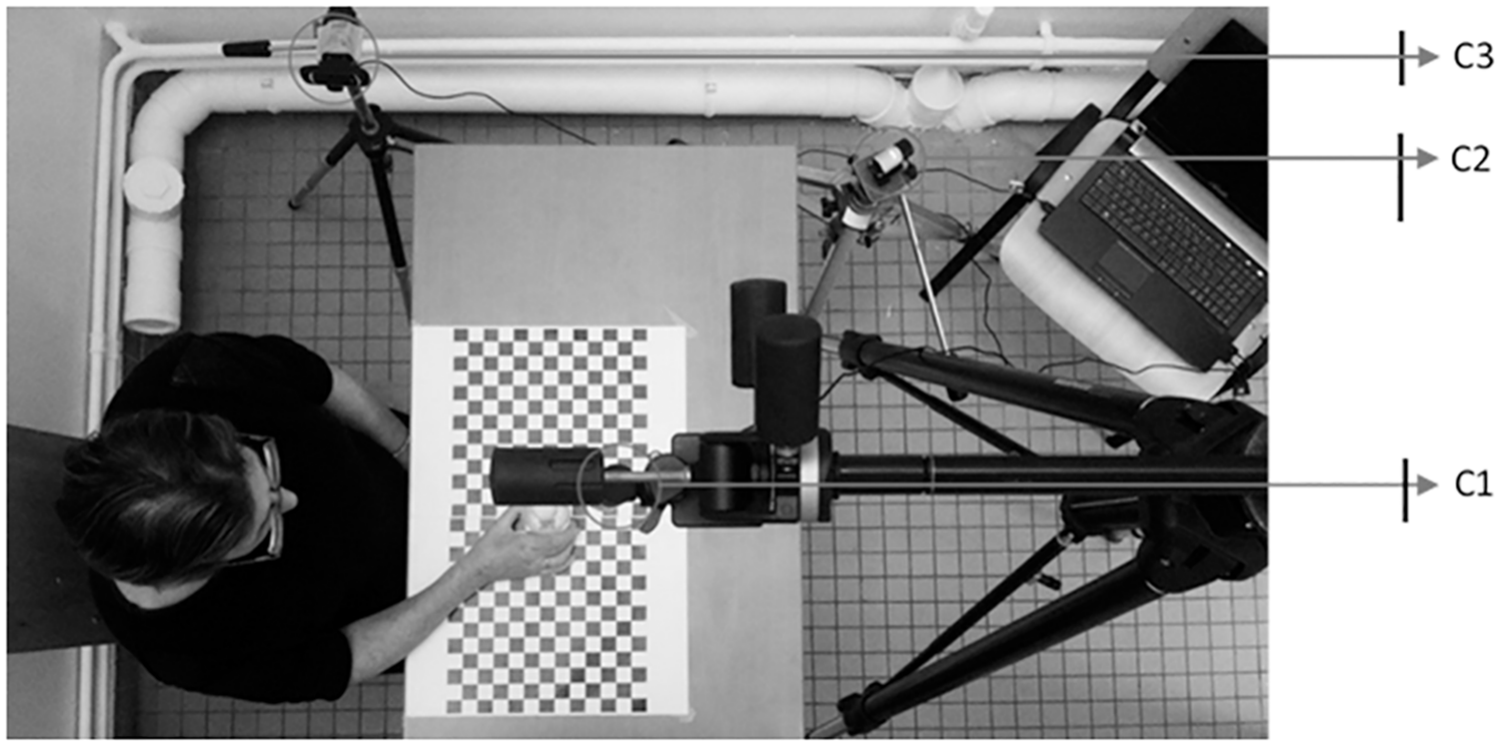
Experimental set-up. The circles indicate the position of the cameras. One camera was positioned directly above the object (80 cm high) (C1), and the other 2 were placed at a height of 75 cm on the contralateral side of the hand being evaluated: one 30° to the sagittal plane (at a distance of 75 cm from the object in the horizontal plane) (C2) and the other 30° behind the frontal plane (at a distance of 70 cm from the object in the horizontal plane) (C3).

The grasping tasks were recorded using 3 web-cameras located around the object (Fig 2). Video clip analysis has been shown to be a valid and reliable tool for the routine clinical evaluation of upper limb function in patients after stroke [38]. The set up ensured a good view of the participant’s hand and fingers during all the grasping tasks [39]. Open source iSPY software was used to activate the 3 cameras simultaneously when the patient began the task.

Three trials were recorded for each object with a ten second pause between each. The order of presentation of the objects was randomized and the instruction given was to: “**Grasp the object as if you are going to use it**”. The objects were presented as shown in Fig 1 i.e. the tissue packet vertically and the spoon in a cup.

Video analysis and rating: The grasps were rated from the videos by two experienced observers (specialist physiotherapists) who independently rated the type and the quality of each grasp. The analysis of the type was performed in two steps. First, one observer noted all the elementary features of each grasp: constitution of the virtual and real fingers (fingers or palm on each side of the opposition axis, use of the thumb), and the parts of the fingers and hand that contacted the object (pulpar, palmar or lateral surface of the distal or proximal phalanxes of the fingers, middle, thenar or hypothenar eminence of the palm). The grasp-types were then defined qualitatively by grouping the elementary features within the whole dataset. The different grasp-types and the description of the criteria are presented in the Results section. Each trial for each patient was then re-examined and classed separately by the two observers. When the result differed between the two observers, they re-analysed the video together until they reached a consensus. The initial inter-rater agreement was about 80%.

A Functional Grasp Scale (FGS) was developed for the purpose of the study to rate the functional quality of each grasp. We based the FSG on a French scale named “Classification Fonctionnelle de la prehension d’Enjalbert” [40]. We considered the scale was not sufficiently detailed for the purposes of this study and therefore we modified it using the results of the study by Bensmail et al [32]. The FGS was rated from 0: unable to grasp to 10: same as healthy subject, details are provided in Table 2. The same grasp-type could be assigned a different score depending on the object. e.g. an interdigital grasp was not functional for the bottle (scoreless or equal to 5) but was functional for the teaspoon (score 6 or higher). Scores were assigned independently by each observer and when differences occurred, discussion allowed a consensus to be reached. The initial inter-rater reliability for the FGS was about 70%.

**Table 2:** Functional Grasp Scale (FGS).

Score	Characteristics
0	Unable to grasp object
1	Grasp not sufficiently stable to lift object
2	Object is grasped and lifted but it falls immediately
3	Object is lifted for several seconds then falls
4	Grasp steady but not adapted to the object shape
5	Grasp adapted to the object shape but not functional
6	Functional grasp but precarious
7	Stable and functional grasp but unable to release object
8	Stable and functional grasp, able to release with some difficulty
9	Stable and functional grasp but different from healthy subjects
10	Stable and functional grasp similar to healthy subjects

### Statistical analysis

The distribution of the grasp-type used for each object was compared between the patients and healthy subjects. A total of 456 grasps were analysed for the patient group (4 objects x 3 trials x 38 patients) and 120 for the control group (4 objects x 3 trials x 10 controls). The distribution of grasp-types was also analysed as a function of the ARAT and FMA-UE scores, independently from the object.

Medians and inter-quartile ranges were calculated for each clinical parameter.

Global grasp quality for each patient was evaluated by the mean FGS score for the 4 objects and 3 trials. Correlation and partial correlation analysis were performed between the mean FGS score and the FMA-UE and ARAT scores. A Mann-Whitney U test was used to compare groups of patients combined with post-hoc analysis.

The analysis of the relationships between clinical data and the FGS score was performed separately for each grasp-type, independently from the object. Since the patients could use different grasps for the same object, only the 3rd trial for each object was entered into the analysis in order to avoid bias (4 x 38 trials). First, a correlation analysis (Pearson’s coefficients) was carried out between each specific clinical impairment and FGS. The variables correlated with FGS (level of significance p<0.05) were then included in a multiple regression model [41] if the number of grasps was > 10. Multiple regression was performed with FGS score as a dependent variable and the significantly correlated clinical variables as independent variables, by using a stepwise procedure with a step by step forward selection. This methodological choice was made in order to determine the main clinical parameters that explained the different grasp-types, independently from the nature of the object.

## Results

### Clinical examination

**Fig 3:**
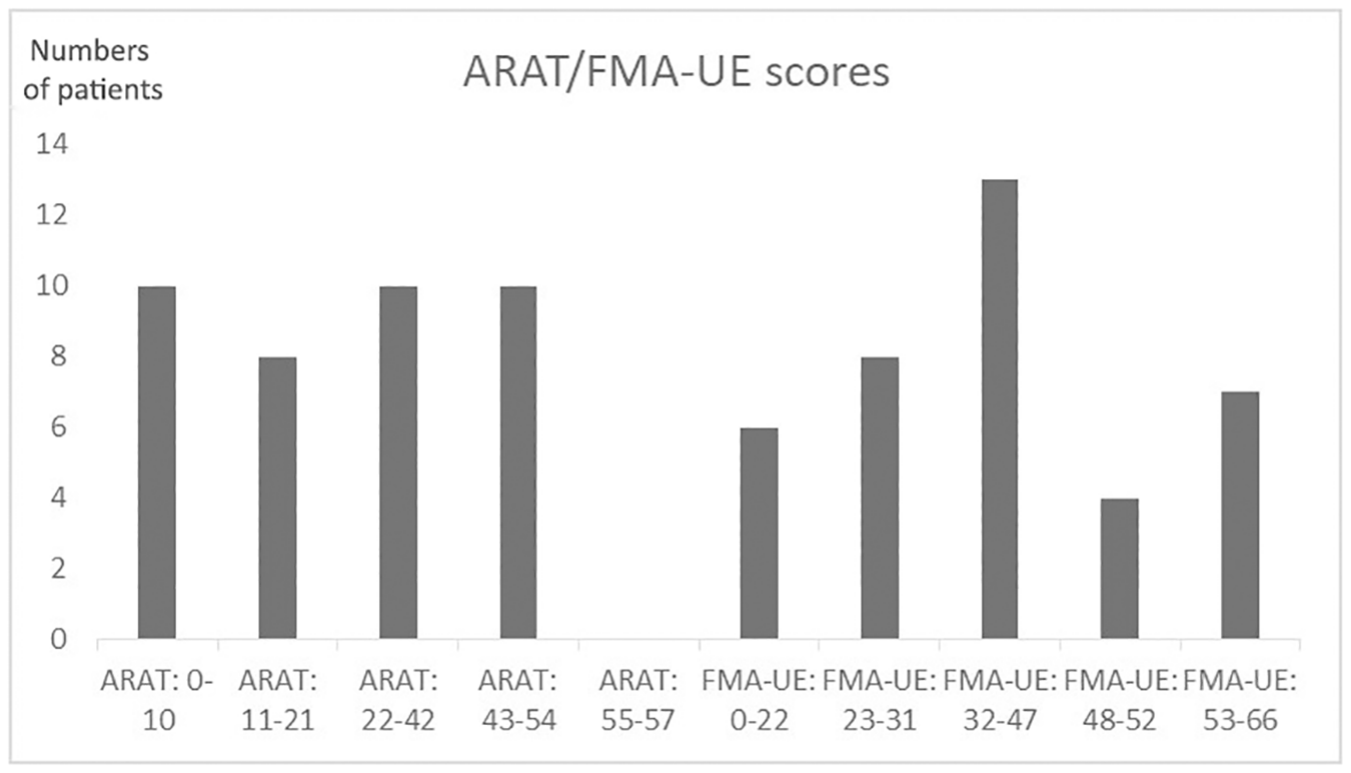
ARAT/FMA-UE scores. The bars represent the number of patients in each score-group for the FMA-UE (Fugl-Meyer Assessment-Upper Extremity) and ARAT (Action Research Arm Test scores). The grouping of scores was based on the study by, Hoonhorst et al [42].

The principal individual results of the clinical examination are presented in S1 Table. The impairments and disability ranged from mild to severe as shown by the ARAT and FMA-UE scores (Fig 3).

### Grasp-types

Eight grasp-types were identified from the whole database of 576 grasps (456 for the patients and 120 for the controls): multi-pulpar, pluri-digital, lateral pinch, palmar, digitopalmar, raking, ulnar grasp and inter-digital. Six of these grasps have already been identified by Bensmail et al [32] and the pluri-digital and digito-palmar grasps were identified in the present study.

**Fig 4:**
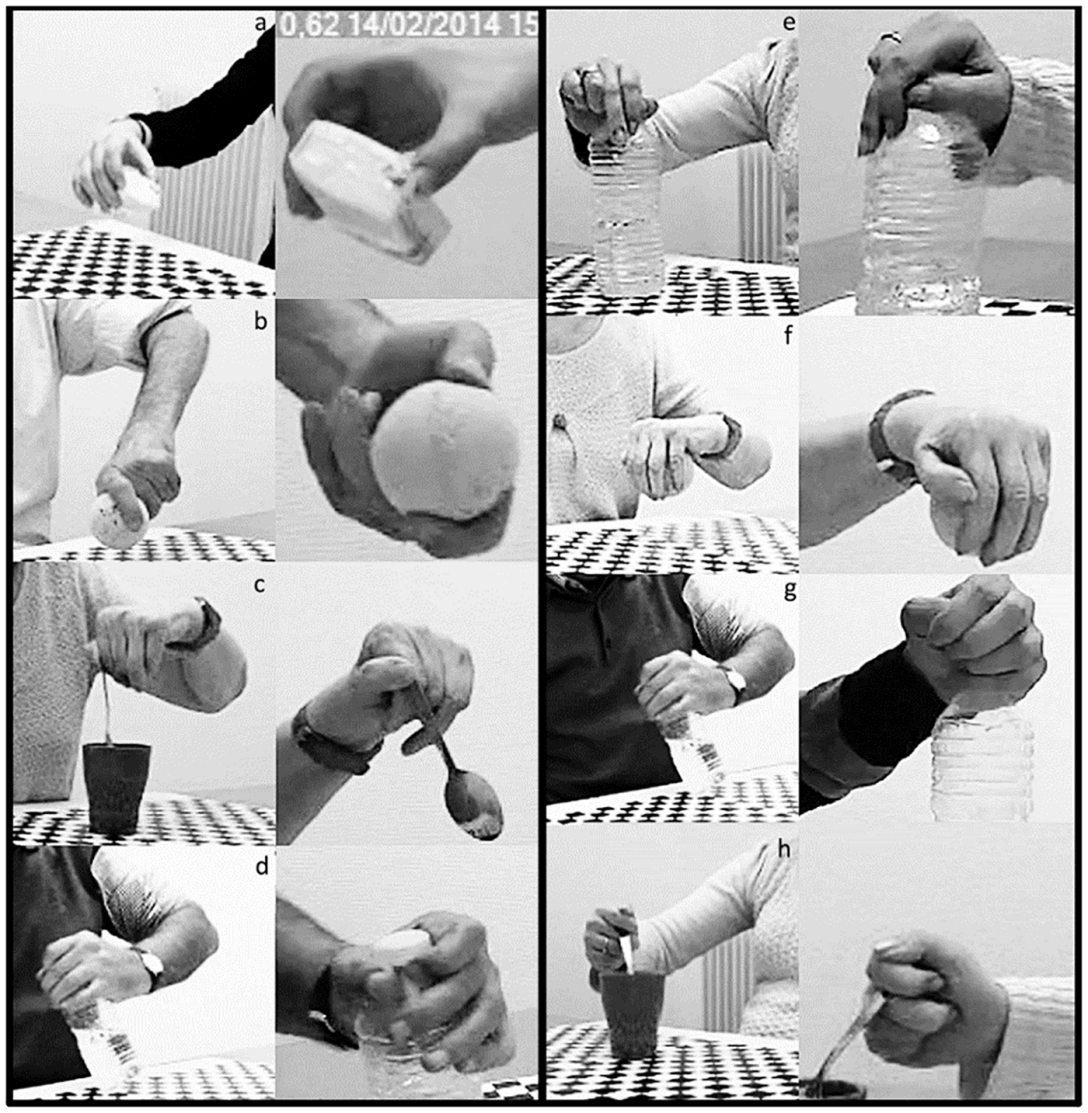
Grasp-types. a: Multi-pulpar, b: Pluri-digital c: Lateral Pinch, d: Palmar, e: Digito-palmar, f: Raking, g: Ulnar, h: Inter-digital.

*Multi-pulpar*: involves the distal pad of any finger, the proximal phalanxes are not involved. The thumb is positioned in opposition to the other fingers and the palm of the hand is not involved (Fig 4a).
*Pluri-digital*: involves the thumb and one or more other fingers (bi-digital, tri-digital, tetra-digital or penta-digital). The proximal phalanxes are involved but the palm is not (Fig 4b)
*Lateral pinch*: involves the pad of the thumb in opposition to the lateral side of the index finger (Fig 4c).
*Palmar*: involves the palm and all the fingers, which wrap around the object. The thumb is in opposition to the other fingers (Fig 4d).
*D**igito-palmar*: involves the palm in opposition to one or several fingers (Fig 4e).
*Raking*: involves the palm and last four fingers. The thumb is not involved (Fig 4f).
*Ulnar*: involves the ulnar side of the palm and the fourth and fifth finger, which are flexed (Fig 4g).
*Inter-digital*: involves the lateral sides of two fingers, adjacent or not. Often the thumb is not involved but sometimes it envelops the object to stabilize the grasp. The palm is not involved (Fig 4h).

### Distribution of grasp- types for each object in both groups

Table 3 summarizes the distribution of the grasp-types used for each object. The healthy subjects used only 4 grasp-types (Multipulpar, Pluri digital, Lateral pinch and Palmar). The other 4 grasp-types (Digito-palmar, Raking, Ulnar and Inter-digital) were used exclusively by the patients. The grasp-types used by the healthy subjects will be named “standard” and the four others “alternative” throughout the rest of the article. Both healthy subjects and patients used pluri-digital and multi-pulpar grasps the most frequently, however the percentage of use differed between groups. The distribution of grasp-types depended on the object for both the stroke and the healthy subjects, however variability of grasp-type for a given object was higher in the stroke group. (Individual results are presented in S2 Table).

**Table 3:** Distribution of the grasp-types across the 456 trials for each object.

	Tissue Packet	Tennis Ball	Water Bottle	Spoon	Total
	Patients/HS	Patients/HS	Patients/HS	Patients/HS	Patients/HS
**Multipulpar**	**28% / 80%**	16% / 20%	10% / 10%	15% / 10%	17% / 30%
**Pluri-digital**	24% / 20%	**25% / 70%**	**30% / 67%**	21% / **50%**	**25% / 52%**
**Lateral-pinch**	0% / 0%	0% / 0%	4% / 0%	**41**% / 40%	11% / 10%
**Palmar**	0% / 0%	3% / 10%	10% / 23%	0% / 0%	4% / 8%
**Digito-palmar**	6% / 0%	7% / 0%	11% / 0%	4% / 0%	7% / 0%
**Raking**	5% / 0%	14% / 0%	5% / 0%	1% / 0%	6% / 0%
**Ulnar**	3% / 0%	0% / 0%	9% / 0%	4% / 0%	4% / 0%
**Interdigital**	0% / 0%	0% / 0%	0% / 0%	12% / 0%	3% / 0%
**Fail**	34% / 0%	34% / 0%	21% / 0%	3% / 0%	23% / 0%

HS: Healthy Subjects. The highest percentages are shown in bold. The grasps are ranked with multipulpar first, then according to their frequency of use in the control group, then in the patient group.

### Distribution of the grasp-types according to level of impairment (FMA-UE score) and functional capacity (ARAT score)

**Fig 5:**
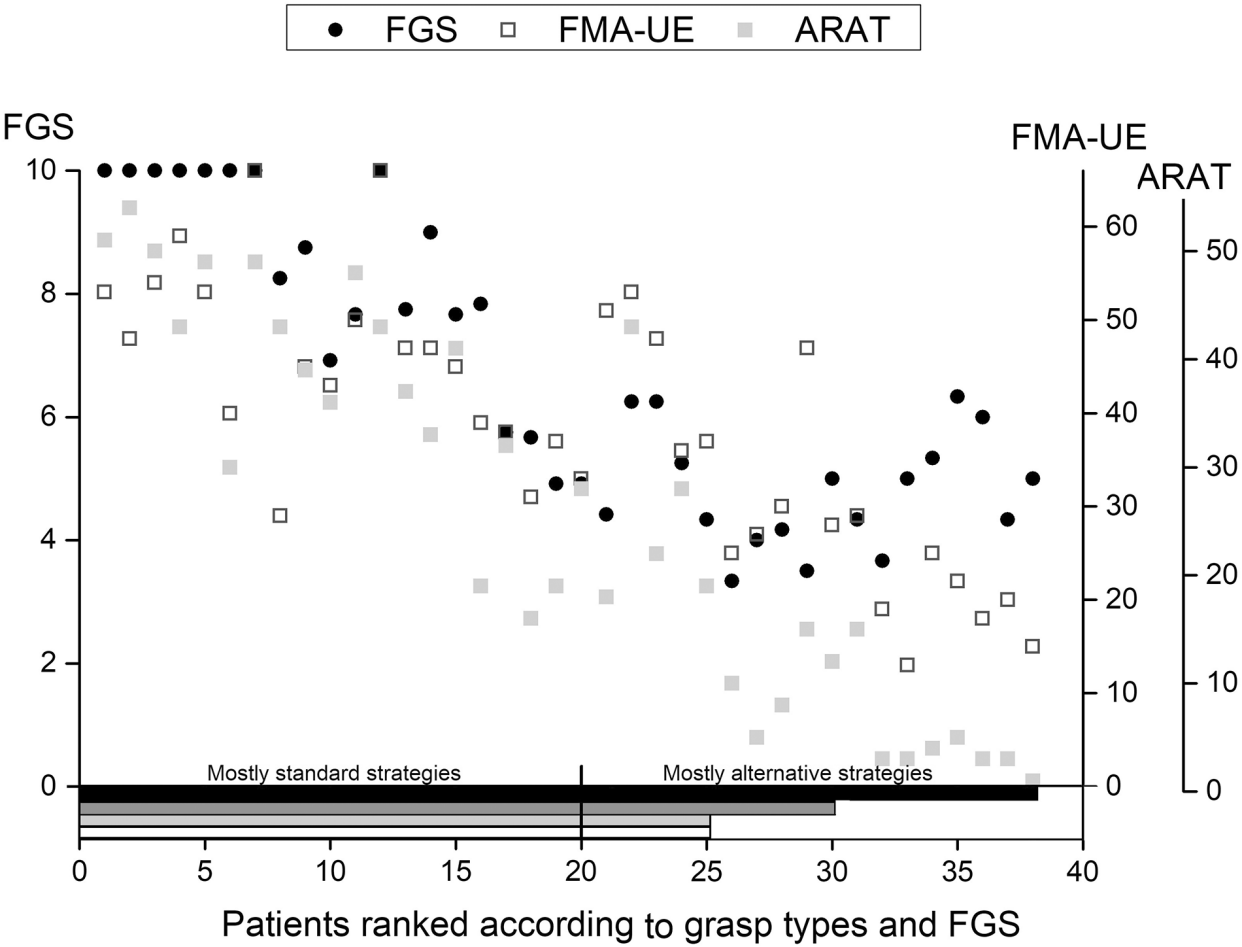
Mean FGS, FMA-UE and ARAT score in individual patients. The patients are ranked according to their grasp type and their FGS score (see S3 Table). Horizontal bars represent the objects the patients were able to grasp (Black: spoon, dark grey: bottle, light grey: ball, white: tissues).

The distribution of the grasp-types across impairment levels (FMA-UE score) was quite variable. They are presented in Fig 5, ranked according to grasp-type for a clearer presentation of the results. The first sorting key was the grasp type (same order as Table 3) the patients used during the last trial for the tissue packet, then the bottle, then the spoon. The second sorting key, for similar grasp types, was mean FGS score for each patient. Individual results of the patients are presented using the same ranking order in Fig 5 and S1 and S2 Tables.

Twenty-five patients with mild (FMA-UE >47) or moderate (FMA-UE 31–47) impairment and functional capacity (ARAT 16–56) were able to grasp all 4 objects. Twenty of them used mostly standard grasp-types. The 5 others rarely used standard grasp-types and instead used alternative grasp-types, mainly digito-palmar and raking. Thirteen patients who had severe impairment and low functional capacity (FMA-UE ≤30 and ARAT ≤15) failed to grasp at least 1 object and mostly used alternative strategies. Five of them (with FMA-UE 25–30) failed to grasp the tennis ball and tissue packet and grasped the bottle and spoon using mainly alternative grasp-types (particularly ulnar for the bottle). The last 8 patients (FMA-UE 13–29, ARAT 0–15) were only able to grasp the spoon (4/8 used an interdigital alternative grasp-type).

The 20 patients who used standard grasp types had significantly better FMA-UE and ARAT scores than the 18 patients who used alternative grasp-types or failed (Mann-Whitney p = 0.0005 and p ≤0.0001 respectively). The ARAT score more clearly represented the choice of grasp-type than the FMA-UE: 13/14 patients with ARAT ≥36 always used standard grasp-types while 22/24 patients with ARAT <36 more frequently used alternative grasp-types.

The choice of a particular grasp-type was not strictly dependant on the level of impairment or functional capacity as measured by the FMA-UE and ARAT, as shown in Fig 5. Patients 21–25 mostly used alternative grasp-types, although they had similar or higher ARAT and FMA-UE scores than patients 15–20 who used mostly standard grasp-types. In addition, most of the patients with severe impairment (patients 26–38) used alternative grasp-types, however they were still partially functional (mean FGS above level 4) despite low FMA-UE and ARAT scores.

### Grasp quality according to level of impairment (FMA-UE score) and functional capacity (ARAT score)

**Fig 6:**
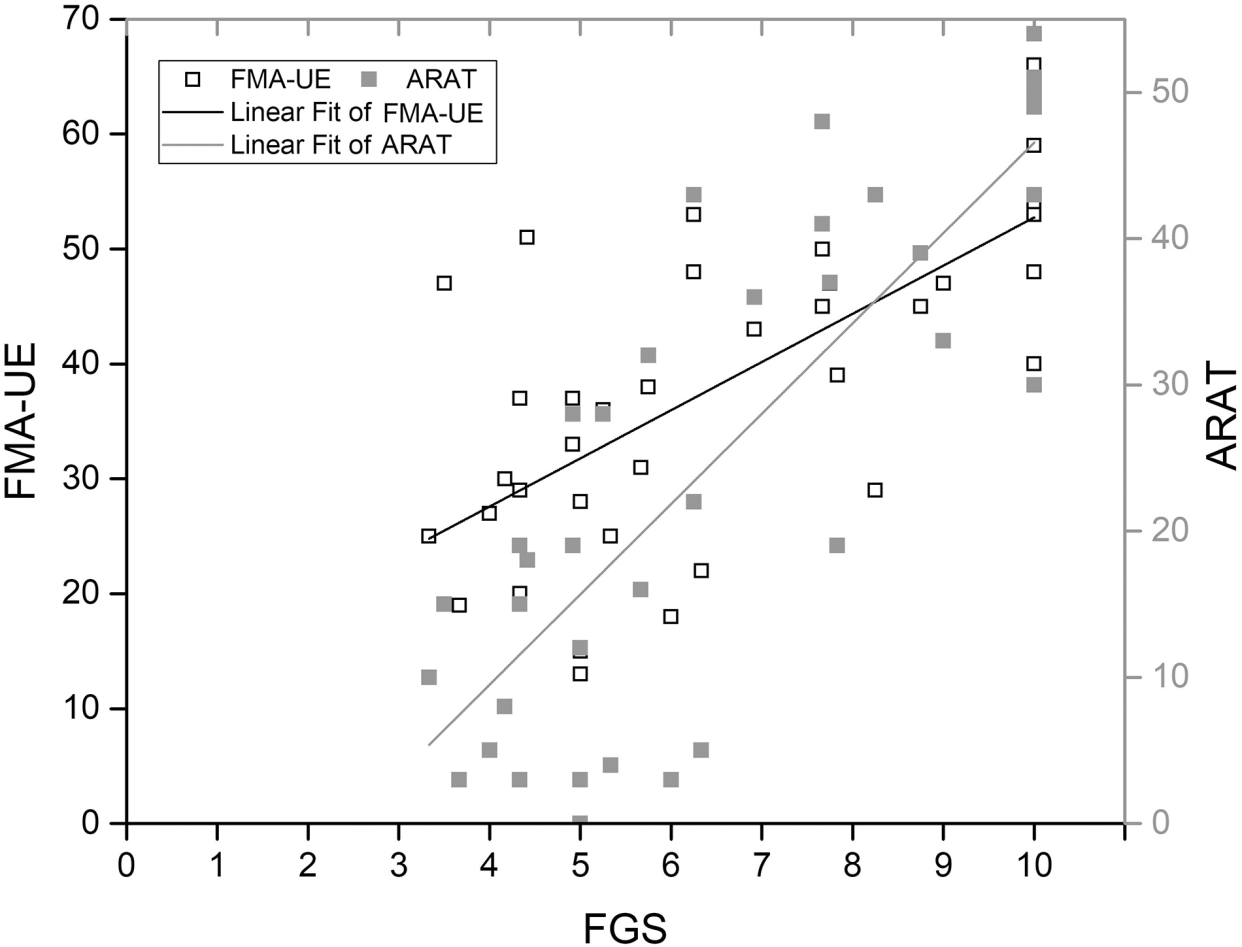
FMA-UE and mean FGS scores as a function of ARAT score.

Mean FGS score was correlated with mean FMA-UE (r = 0.688) and mean ARAT (r = 0.822) scores; and the FMA-UE and ARAT scores were also correlated (r = 0.837) (Fig 6). A partial correlation analysis showed that the correlation between mean FGS and FMA-UE disappeared for a given ARAT level, while the correlation between mean FGS and ARAT remained (r = 0.620) for a given FMA-UE level.

#### Clinical determinants of grasp quality (FGS) for each grasp-type

Correlations between clinical parameters and grasp-types and multiple regression analyses were performed on the last trial for each object (n = 118 successful trials)

Correlations between clinical parameters and Functional Grasp Scale: The principal results of the significant Pearson’s correlations between clinical parameters and quality of the grasp for each grasp-type are presented in S3 Table. The grasp-type was mainly correlated with MRC (strength) and MAS (spasticity) scores. In contrast, loss of sensation was poorly correlated with most grasp-types.

There were no significant correlations between clinical parameters and Functional Grasp Scale score for the interdigital grasp.

Stepwise regression: Stepwise regression analysis was performed separately for the grasp-types that were used in at least 10 trials, independently from the object. The clinical parameters significantly correlated with the FGS were entered into the analysis as independent variables and FGS as a dependent variable.

**Fig 7:**
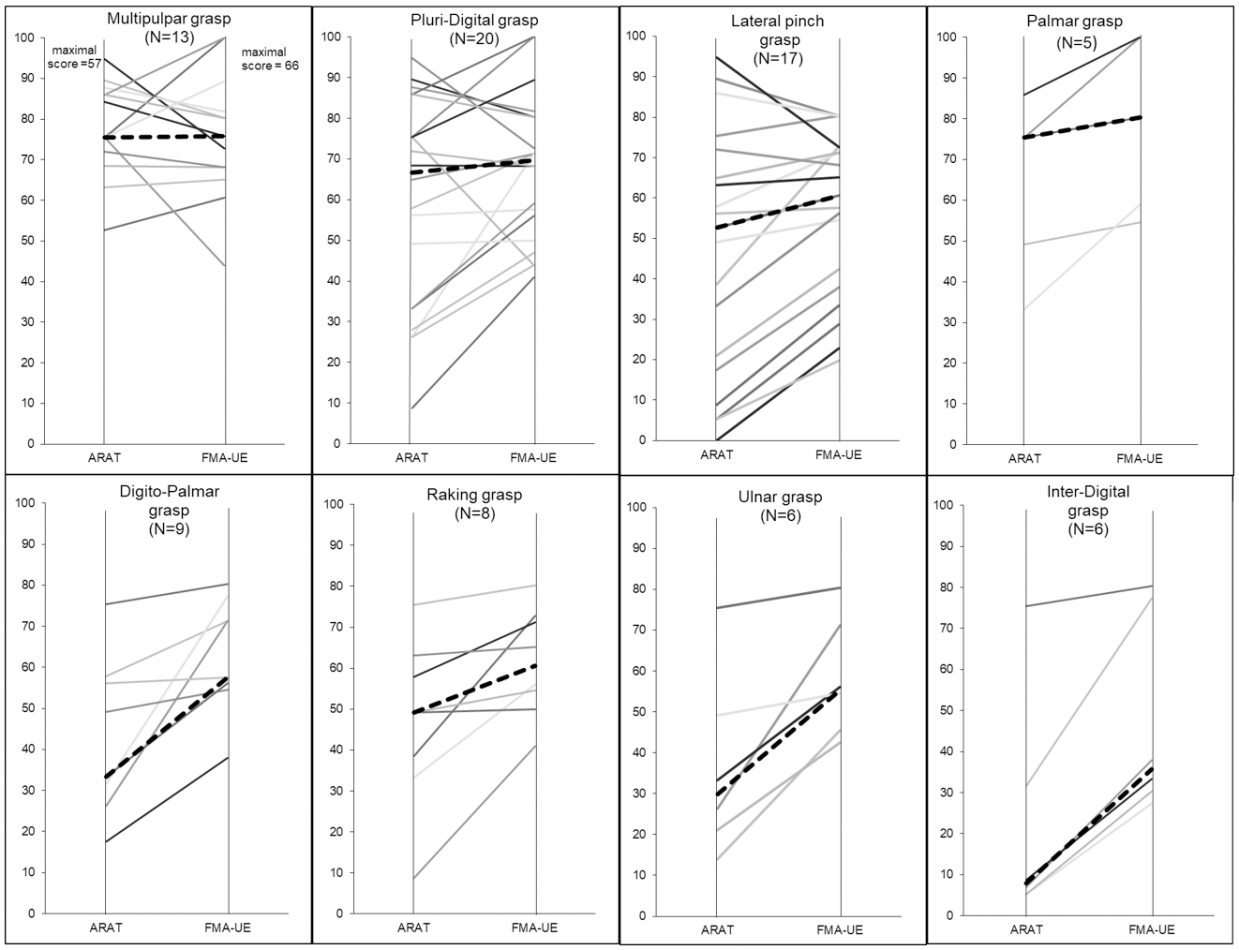
ARAT and FMA-UE scores of individual patients for each grasp-type. In bold the median ARAT and FMA-UE scores. For clarity, ARAT and FMA-UE scores have been normalised out of 100.

Pluri-digital grasp: Forty-one movements were inputted in the model from the 20 patients who used a pluri-digital grasp at least once. The FMA-UE scores ranged from 27–66 and ARAT from 5–54, median: 46 and 38 respectively (Fig 7b). MRC elbow extensor score *(p = 0*.*01)* and finger extensor score *(p = 0*.*01)* were selected, explaining 80% of the variance of the FGS.

FGS Pluri-digital grasp = -3.23 + 1.2 MRC elbow extensors + 1.35 MRC finger extensors

Lateral-pinch grasp: Nineteen movements were inputted in the model from the 17 patients who used a lateral-pinch grasp at least once. The FMA-UE scores ranged from 13–53 and the ARAT from 0–54, median: 30 and 40 respectively (Fig 7c). Side of hemiparesis *(p = 0*.*00)*, MRC thumb extensor score *(p = 0*.*03)* and wrist flexor score *(p = 0*.*03)* were selected, explaining 90% of the variance of the FGS.

FGS Lateral-pinch grasp = 13.55 - 3.01 side of lesion + 1.38 MRC thumb extensor score + 0.8 MRC wrist flexor score

Digito-palmar grasp: Ten movements were inputted in the model from the 9 patients who used a digito-palmar grasp. The FMA-UE scores ranged from 25–53 and ARAT from 10–43, median 38 and 19 respectively (Fig 7d). Each one of the 4 objects was grasped at least once with this grasp-type. MAS thumb adductor score *(p = 0*.*02)* was selected, explaining 74% of the variance of the FGS.

FGS Digito-palmar grasp = 3.73 - 2.6 MAS thumb adductor score

Raking grasp: Eleven movements were inputted in the model from the 8 patients who used a raking grasp. The FMA-UE scores ranged from 27–53 and the ARAT from 5–43, median 40 and 28 respectively (Fig 7e). The MRC wrist flexor score *(p = 0*.*02)* was selected, explaining 74% of the variance of the FGS

FGS Raking grasp = 3,93 + 2.25 MRC wrist flexor score

## General discussion

This study evaluated the different grasp-types used by patients with stroke and healthy subjects for 4 objects frequently used in daily life activities. As expected, both groups used different grasp-types depending on the object and several different grasp-types could be used for the same object.

The control group used four “standard” grasp-types. The stroke group also used these grasp-types but with a higher degree of variability and they also used alternative grasp-types. In addition, the grasp-types used by the patients with stroke were related to the global level of disability and also to specific clinical impairments, in particular strength and spasticity (MRC and MAS scores).

Only one other study has carried out an analysis similar to the present study [32]. Six of the seven grasp-types found in their study were also found in the present study (*Multi-pulpar*, *Ulnar*, *Palmar*, *Lateral Pinch*, *Inter-digital* and *Raking*), suggesting that they are particularly common in patients with stroke. However, the *intrinsic* grasp was not found in the present study, likely because of differences in the objects used. Two additional grasp-types were found in the present study, the *Pluri-Digital* and the *Digito-Palmar* grasps, used by the patients with stroke. This difference can also likely be attributed to the nature of objects used [3,5,6,43] as well as the larger sample size of the present study.

### Distribution of grasp-types in the patients and healthy subjects

The healthy subjects used four standard grasp-types. The multipulpar and palmar grasps correspond respectively to the precision grip and power grasp described by Napier; they were consistently used to respectively grasp the lighter (tissues, 80%) and heavier (bottle, 23%) objects. The lateral pinch corresponds to the intermediate “key grip” described by Iberall (1986). This grasp-type was exclusively used to grasp the spoon (40%), which is consistent with the flat shape of that object. In addition to these three classical categories of grasp, the pluri-digital grip, a type of power grasp [31], was preferred for the bottle (67%), the ball (70%) and the spoon (50%). This surprising finding may be due to the fact the tasks only involved grasping the object, and not carrying out a specific activity with the object. It is consistent with the finding that small and lightweight objects are not necessarily grasped with precision-type grips during functional tasks [31].

The grasp-types used by the patients with stroke were more variable. This is in accordance with Lang et al [20] who found that, in general, intra- and inter-individual variability of grasp movements is increased in the affected hand. The patients in the stroke group used precision (multipulpar), intermediate (lateral pinch) or power grasps (palmar and pluri-digital) for the same objects as the healthy subjects, but less frequently. In the other cases, they failed or used alternative grasp strategies never used by the healthy subjects. Digito palmar, Ulnar and Raking grasps could be considered as compensatory alternative strategies for grasping relatively large objects, and the Interdigital grasp as a compensatory alternative strategy for lateral pinch or precision grasp for a small object. The common characteristic of these alternative strategies is that they do not involve the thumb.

The choice of standard or alternative grasping strategies globally depended on the FMA-UE score, suggesting that the patients preferentially, but not systematically, used standard grasp types if they could. However, there was no clear-cut limit, suggesting that more severe impairment decreases the choice of available grasp-types, and the final choice is determined by the specific impairments of each individual patient. The ARAT score better accounted for the choice of standard or alternative grasp type since patients who regularly used standard grasp types had ARAT scores ≥36. In addition, the ARAT was correlated to the grasp quality (FGS), independently from the FMA-UE. This close relationship is not surprising since the FSG and ARAT are both functional scales and that the instruction given to the patient was: “Grasp the object as if you are going to use it”. The functional quality of a grasp may be the result of the acquisition of new, alternative, skilled behaviour by some patients, relatively independently from their neurological impairment. Another hypothesis is that the critical limit for use of standard grasp types is thumb opposition, which probably affects ARAT score more than FMA-UE (only 1 item). This indirectly suggests that treatment should aim to improve active thumb movement, either by decreasing spasticity or by improving muscle strength and control, to facilitate progression from an alternative grasp to a functional one.

It is surprising that the patients used precision grips since it is well known that corticospinal tract lesions affect precision grip [44,45] because of the reduction in independence of the fingers and thumb [46]. The precision grip is specific to humans and higher primates [47,48], and is controlled by the recently evolved cortico-motoneuronal spinal tract [49,50]. The alternative grasping strategies used by patients are similar to those of less evolved monkeys (e.g. *squirrel monkeys*) that have no thumb opposition and less developed cortico-spinal tracts [51]. The preservation of precision grasps in some patients might be attributed to less severe lesions of the pyramidal tract or recovery of good function due to cortical plasticity, as suggested by the relatively good FMA-UE and ARAT scores (median 50/66 and 43/57 respectively). The pattern of alternative strategies could be due to Jackson’s dissolution phenomenon [52,53]. A lesion of the phylogenetically recent cortico-motoneuronal pathway perturbs the most evolved behaviours, inducing the return of older, less evolved behaviours. This hierarchical concept of motor control is also consistent with Bernstein’s view on dexterity [54].

Another (not incompatible) explanation could be that the choice of strategy depends on the affordances of the object i.e. the relationship between the shape of the graspable parts of the object and the capacity of the hand [55,56,57]. Healthy subjects grasp the bottle at the base, which is more convenient for drinking, while patients grasp it at the top because they are unable to open their hands sufficiently to grasp the base. The shapes of the bottle and spoon enable different possible grasp-types that only require a small amount of hand opening. In contrast, the relatively uniform shape and the size of ball and tissue packet, imposes larger hand opening, explaining the large number of failures (34%) for these objects in patients who may have a limited hand aperture.

### Relationships between clinical parameters and grasp quality

The clinical factors which explained the quality of each grasping will be discussed separately for each grasp-type.

#### Multi-pulpar grasp

It was interesting that most of the 25 patients who used a multi-pulpar grasp (which belongs to the precision class) had high ARAT scores (median 43) (Fig 7), probably because the use of this type of precision grasp requires some preservation of the pyramidal tract and more complex motor skills. There were no significant variables that explained the relationship between the clinical parameters and the quality of the multi-pulpar grasp in the linear regression model. This likely suggests that many clinical parameters influence this grasp-type, and their combination allows some patients to use this grasp. Spasticity of the wrist flexor muscles had a negative influence on the quality of multi-pulpar grasp. Thus treatment to reduce spasticity in these muscles, such as botulinum toxin injection, might be relevant for these patients, enabling them to use this grasp and improve hand motor function.

#### Pluri-digital grasp

The pluri-digital grasp was the most frequently used by patients, but was used half as often as the healthy subjects. It was used by 21 patients who had very varying levels of impairment and functional capacity (Fig 7). In most cases, the pluri-digital grasp was used by patients with good to moderate recovery (FMA-UE and ARAT scores) consistently with Hoonhorst [42]. More surprisingly, it was also used by 3 patients with a low functional ability (ARAT 5–15) and variable motor capacity (FMA-UE 27–47) specifically to grasp the spoon. These patients had severe impairment (FMA-UE 27, 29) or, in one case, moderate impairment (FMA-UE: 47) associated with severe hand spasticity. These 3 patients managed to passively wedge the spoon between the thumb and fingers despite their low level of distal function, ensuring a stable but poorly functional grasp (FGS 4 to 6).

The quality of the pluri-digital grasp was explained by the MRC of the finger extensors and elbow extensors, which explained 80% of the variance of FGS score. This suggests that an efficient pluri-digital grasp requires some independent movement of the fingers and hand opening ability, consistent with Jeannerod M [3,58]. The correlation with the elbow extensor MRC score illustrates the close relationship between reaching and grasping. It is likely that a relatively good control of elbow motion is necessary to position and orientate the hand appropriately to grasp the object using a pluri-digital grasp. This suggests that to improve this grasp, treatment should focus on exercises to improve finger extension.

#### Lateral pinch grasp

The lateral pinch grasp was used by 19 patients with a wide range of impairments and functional abilities (median FMA-UE 40 and ARAT 30) (Fig 7). Similarly to the healthy subjects, it was most often used to grasp the spoon. Ninety percent of the variance of FGS score for this grasp was explained by a negative correlation with the side of the lesion and by a positive correlation with the MRC score of the thumb extensors and wrist flexors. Further analysis of the data showed that patients with right-sided hemiparesis who used the lateral pinch grasp had higher FGS scores than those with left-sided hemiparesis. The reason for this difference is uncertain and there are no elements from which to draw any conclusion. Not surprisingly, thumb extension is important for this grasp-type. The role of the wrist flexor muscles may be to facilitate opening of the fingers via a voluntary tenodesis effect [59]. It is likely that patients with higher levels of recovery use the thumb extensor muscles, while those with less recovery use the wrist flexor muscles as a compensatory mechanism to passively extend the thumb and fingers and grasp the object.

The strong correlations between FGS score and strength of the proximal muscles probably reflects the role of these muscles in the precise positioning and orientating of the hand in order to grasp the spoon.

Importantly for clinical practice, these results suggest that treatment to reduce spasticity of the wrist flexor muscles, if present, could reduce the capacity to use a lateral pinch grasp. Treatment should focus on active finger and thumb extension.

#### Palmar grasp

Only 5 patients, with a relatively moderate impairment (median ARAT score 43 and FMA-UE 53) (Fig 7), used a palmar grasp, probably because it requires relatively large opening of the hand. The number of patients was too low for stepwise regression analysis to be performed, however the correlation analysis showed that spasticity of the arm and hand muscles could have a negative influence on the quality of the palmar grasp. Treatment to reduce spasticity of the arm and hand muscles might therefore improve this grasp and thus functional capacity of patients after stroke. In addition, the correlation analysis showed that elbow flexion ROM and supination strength were necessary to ensure a good quality palmar grasp. This indicates that in patients with spasticity of the pronator muscle, treatment to reduce this spasticity would be pertinent.

#### Digito-palmar grasp

The digito-palmar grasp was the most frequently used alternative grasping strategy in 7 patients with relatively low ARAT and FMA-UE scores (median ARAT 19 and FMA-UE- 38) (Fig 7). Seventy-four percent of the variance of the FGS for this grasp was negatively explained by the MAS score of the thumb adductor muscles. This suggests that patients who used a digito-palmar grasp tried to use their thumb and were limited by spasticity of the thumb adductors. Thus it would seem that the digito-palmar grasp is used as a compensatory strategy when the impairment of thumb movement impedes the use of more efficient grasps (e.g. multi-pulpar or pluri-digital). Treatment to reduce spasticity of the thumb and intrinsic finger muscles could allow patients to use a standard grasp that would be more adapted to the object.

#### Raking grasp

The raking grasp was used by 8 patients with a low clinical level (median ARAT 28 and FMA-UE-UE 40) (Fig 7). It was mainly used to grasp the larger objects (tissue packet and tennis ball). It could be a strategy to compensate for impairment of finger movement associated with a pathological flexor synergy of elbow flexion and pronation that compromises use of a palmar grasp. The synchronous elbow flexion and supination that is necessary for a palmar grasp is not necessary for raking. Seventy-four percent of the variance of this grasp was explained by the MRC score of the wrist flexor muscles. The action of the wrist flexors is probably to facilitate passive opening of the fingers using the tenodesis effect [59] and therefore to compensate the impairment of the finger extensors. Then, the object can be stabilized between the palm and fingers. The hand function of these patients may be improved by treatment to reduce spasticity in finger flexors muscles in order to reduce pathological finger synergies that inhibit effective grasps.

#### Ulnar grasp

The ulnar grasp was used by only 6 patients with low ARAT and FMA-UE scores (median ARAT 17 and FME-UE 36) (Fig 7). This suggests that this grasp is a compensatory strategy only used by patients with severe impairment that impedes the use of more complex grasps, including raking. Probably the ulnar grasp is associated with a poor recovery of thumb extensor muscles. We suggest that training programs to improve thumb movement in this group of patients may allow use of other standard grasps (such as lateral pinch), which are more adapted to grasp an object.

#### Inter-digital grasp

This grasp was used by 5 patients with poor recovery (median ARAT 4 and FMA-UE 23) (Fig 7) to grasp the spoon. There were no significant correlations between FGS score and any clinical parameters. The inter-digital grasp is an alternative grasp-type [27], which could be used by patients with very little functional upper limb capacity probably because it results in a stable grasp using only the passive properties (shape, stiffness and friction) of the fingers.

These results illustrate how specific clinical features of impaired motor control influenced the choice of a given grasp and explained its quality. The patients who had a moderate or relatively good functional ability were able to use similar grasp strategies to the healthy subjects. This was conditioned by sufficient finger and elbow extensor strength (pluri-digital grasp); thumb extensor and wrist flexor strength (lateral pinch) and forearm supinator strength (palmar grasp). Spasticity of the arm and hand muscles reduced the quality of the standard multi-pulpar and palmar grasps and also the digito-palmar grasp. This may therefore be pertinent to treat, for example using botulinum toxin injections, in patients who use these grasps.

By contrast, the patients who had severe impairment used alternative strategies that did not involve the thumb. These strategies likely compensate specific impairments, as suggested by the results of the correlation and regression analyses. We propose the following hypotheses: 1) the digito-palmar grasp is used by patients who are unable to use pluri-digital or multi-pulpar grasps (because of severe impairment of finger movement); 2) the raking, ulnar and interdigital grasps are probably archaic strategies that require little descending control; 3) the raking grasp could substitute for the palmar grasp when elbow flexion, supination and hand opening are impaired, but it is probably conditioned by the function of the wrist flexors necessary for compensatory behaviour (tenodesis effect to open hand); 4) the ulnar and interdigital grasps could compensate for severely impaired active distal control that prevents use of a lateral pinch grasp (because of severe impairment of thumb abduction). The relationships between hand and upper-limb impairments and compensatory strategies that patients develop are complex. Impairment of hand movement may influence the choice of a particular grasping strategy, which itself constrains the hand orientation for grasping and imposes an alternative direction of the reaching movement. Equally, impairment of shoulder-elbow-wrist coordination probably constrains the grasp strategy by altering the control of hand orientation [14].

Surprisingly, sensation was poorly related to the choice of grasp type. Other authors have reported that relationships between somatosensory impairments and reaching performance vary depending on the magnitude of sensory loss [60] and motor dysfunction [61,62]. In the present study, sensation and proprioception were measured using common clinical tests; however these tests lack precision which limits the conclusions that can be made about the relationships between somatosensory deficits and grasping performance [63].

These results have also important implications for clinical practice and management as they provide some indications regarding the management of patient with stroke depending on their grasp strategies. The precise relationship between spasticity and motor performance is still debated [64,32, 65,66]. Our results suggest that weakness has a greater influence over grasp strategy than spasticity. This would imply that treatment should focus on improving hand strength and control although reducing spasticity may be useful in some cases.

The identification of grasp-types in the present study was an essential first step prior to carrying out a more detailed kinematic analysis (glove or motion analysis) and using new methods of classification of grasp strategies such as deep learning.

## Conclusion

This is the first study to attempt to classify grasp-types in patients with stroke and to suggest appropriate treatments depending on the types of grasps used by individual patients. Two principal classes of grasp were identified: *Standard Grasps*, used by healthy subjects and by patients with higher motor and functional capacities, and *Alternative Grasps* that were never used by healthy subjects, but used more often by patients with lower motor and functional capacities. The results showed that the use of standard or alternative grasps depended on the level of motor and functional recovery of the patient. Regression analysis revealed that the most important determinant of grasp quality was strength, followed by spasticity. This suggests that appropriate treatment to improve hand motor function should combine task-oriented strength training with treatment to reduce spasticity, depending on the grasp-types used by individual patients.

Moreover, it is essential to correctly interpret the grasp-types used by patients with stroke in order to determine if the underlying strategy is based on impairment or compensation [67] since this determines the treatment required.

Longitudinal studies evaluating changes in grasps with recovery will provide greater understanding of the strategies used by patients. Equally, studies evaluating the effects of treatments based on the suggestions provided in the present study will confirm the hypotheses emitted regarding the factors that determine each grasp-type.

## Limitations

The small sample of patients included in this study limits generalisation of the findings.

The FGS was custom-designed for this study and its validity and reliability have not been formally evaluated yet. The results should thus be considered with this in mind. However, the agreement was 70% between two specialised physiotherapists who rated the grasps independently, which we consider to be good.

## Supporting information

S1 Text Functional test used in clinical rehabilitation. (PDF)

S1 Table Principal results of the clinical examination for each subject. (XLSX)

S2 Table Distribution of grasp-types for each object. (XLSX)

S3 Table Principal results of the significant Pearson’s correlations. (TIF)

## References

[1] Pellerin C, Mauget Y., Bouju A., Rouanet F., Petitjean M.E., Dabadie P.. Accident vasculaire cérébral. Médecine d’urgence; 2003 pp. 107–117.

[2] Twitchell TE. The restoration of motor function following hemiplegia in man. *Brain*. 1951; 443–480. 14895765 10.1093/brain/74.4.443

[3] Jeannerod M. The neural and behavioral organization of goal-directed movements. Oxford: Clarendon Press; 1988.

[4] Paulignan Y, Jeannerod M, MacKenzie C, Marteniuk R. Selective perturbation of visual input during prehension movements. 2. The effects of changing object size. *Exp Brain Res*; 1991; 87(2): 407–20. 1769391 10.1007/BF00231858

[5] Santello M, Flanders M, Soechting JF. Postural hand synergies for tool use. *The Journal of Neuroscience*; 1998; 18(23):10105–10115. 9822764 10.1523/JNEUROSCI.18-23-10105.1998 PMC6793309

[6] Mason DR, Gomez JE, Ebner TJ. Hand synergies during reach-to-grasp. *Journal of Neurophysiology*; 2001; 86:2896–2910. 11731546 10.1152/jn.2001.86.6.2896

[7] Santello M, Bianchi M, Gabiccini M, Ricciardi E, Salvietti G, Bicchi A et al Hand synergies: Integration of robotics and neuroscience for understanding the control of biological and artificial hands. *Physics of Life Reviews*; 2016; 17:1–23. doi: 10.1016/j.plrev.2016.02.001 26923030 10.1016/j.plrev.2016.02.001 PMC5839666

[8] Ansuini C, Santello M, Massaccesi S, Castiello U. Effects of end-goal on hand shaping. *Journal of Neurophysiology*; 2006; 95:2456–2465. doi: 10.1152/jn.01107.2005 16381806 10.1152/jn.01107.2005

[9] Bullock IM, Raymond R, Aaron M. A hand-centric classification of human and robot dexterous manipulation. *IEEE*; 2013; 6(2):129–144. 10.1109/TOH.2012.53 24808298

[10] Rosenbaum DA, Vaughan J, Barnes HJ, Jorgensen MJ. Time course of movement planning: selection of handgrips for object manipulation. *Journal of Experimental Psychology*; 1992; 18(5):1058–1073. 1402710 10.1037//0278-7393.18.5.1058

[11] Rosenbaum DA, Chapman KM, Weigelt M, Weiss DJ, van der Wel R. Cognition, action and object manipulation. *Psychological Bulletin*; 2012; 138(5):924–946. doi: 10.1037/a0027839 22448912 10.1037/a0027839 PMC3389205

[12] Sartori L, Straulino E, Castiello U. How objects are grasped: The interplay between affordances and end-goals. *PLos ONE*; 2011; 6(9):e2503. 10.1371/journal.pone.0025203 PMC3182194 21980396

[13] Wing AM, Lough S, Turton A, Fraser C, Jenner JR. Recovery of elbow function in voluntary positioning of the hand following hemiplegia due to stroke. *J Neurol Neurosurg Psychiatry*; 1990; 53(2):126–34. 2313299 10.1136/jnnp.53.2.126 PMC487952

[14] Roby-Brami A, Jacobs S, Bennis N, Levin MF. Hand orientation for grasping and arm joint rotation patterns in healthy subjects and hemiparetic stroke patients. *Brain Research*; 2003; 18;969(1–2):217–29. 12676382 10.1016/s0006-8993(03)02334-5

[15] Levin MF. Interjoint coordination during pointing movements is disrupted in spastic hemiparesis. *Brain*; 1996; 119(1):281–93. 8624689 10.1093/brain/119.1.281

[16] Rohrer B, Fasoli S, Krebs HI, Hughes R, Volpe B, Hogan N et al Movement smoothness changes during stroke recovery. *The Journal of Neuroscience*; 2002; 22(18):8297–8304. 12223584 10.1523/JNEUROSCI.22-18-08297.2002 PMC6758113

[17] Sangole AP, Levin MF. Palmar arch modulation in patients with hemiparesis after a stroke. *Experimental Brain Research*; 2009; 199(1):59–70. doi: 10.1007/s00221-009-1972-5 19690845 10.1007/s00221-009-1972-5

[18] Kamper DG, McKenna-Cole AN, Reinkensmeyer DJ. Alterations in reaching after stroke and their relation to movement direction and impairment severity. *Archives of Physical Medecine and Rehabilitation*; 2002; 83(5):702–7. 10.1053/apmr.2002.32446 11994811

[19] Trombly CA, Quintana LA. The effects of exercise on finger extension of CVA patients. *Am J Occup Ther*; 1983; 37(3):195–202. 6846482 10.5014/ajot.37.3.195

[20] Lang CE, Wagner JM, Bastian AJ, Hu Q, Edwards DF, Dromerick AW et al Deficits in grasp versus reach during acute hemiparesis. *Experimental Brain Research*; 2005; 166(1):126–36. doi: 10.1007/s00221-005-2350-6 16021431 10.1007/s00221-005-2350-6

[21] Raghavan P, Krakauer JW, Gordon AM. Impaired anticipatory control of fingertip forces in patients with a pure motor or sensorimotor lacunar syndrome. *Brain*; 2006; 129(6):1415–25. 16597653 10.1093/brain/awl070 PMC2093998

[22] Johansson RS, Westling G. Coordinated isometric muscle commands adequately and erroneously programmed for the weight during lifting task with precision grip. *Experimental Brain Research*; 1988; 71(1):59–71. 3416958 10.1007/BF00247522

[23] Flanagan JR, Bowman MC, Johansson RS. Control strategies in object manipulation tasks. *Current Opinion in Neurobiology*; 2006; 16(6):650–9. doi: 10.1016/j.conb.2006.10.005 17084619 10.1016/j.conb.2006.10.005

[24] Hermsdörfer J, Hagl E, Marquardt C et al Grip force control during object manipulation in cerebral stroke. *Clinical Neurophysiology*; 2003; 114(5):915–29 12738439 10.1016/s1388-2457(03)00042-7

[25] Nowak DA, Grefkes C, Fink GR et al Dexterity is impaired at both hands following unilateral subcortical middle cerebral artery stroke. *The European Journal of Neuroscience*; 2007; 25(10):3173–84. doi: 10.1111/j.1460-9568.2007.05551.x 17561831 10.1111/j.1460-9568.2007.05551.x

[26] Wenzelburger R, Kopper F, Frenzel A, Stolze H, Klebe S, Deuschl G et al Hand coordination following capsular stroke. *Brain*; 2005; 128(1):64–74. 15471902 10.1093/brain/awh317

[27] Kapandji IA. The upper limb, logistic support of the hand. *Annales de Chirurgie*; 1980; 31(12):1021–30. 610575

[28] Napier JR. The prehensile movements of the human hand. *J Bonne and Joint Surg*; 1956; 902–913. 10.1302/0301-620X.38B4.902 13376678

[29] Iberall T. The representation of objects for grasping. Proceedings of the Eighth Cognitive Society Conference; 1986; 547–561.

[30] Cutkosky MR, Howe RD. Human grasp choice and robotic grasp analysis. *Dextrous Robot Hands*; 1990; 1:5–31.

[31] Feix T, Bullock IM, Dollar AM. Analysis of human grasping behavior: correlating tasks, objects and grasps. *IEEE*; 2014; 7(4):430–41. 10.1109/TOH.2014.2326867 25532148

[32] Bensmail D, Robertson J, Fermanian C, Roby-Brami A et al Botulinum toxin to treat upper-limb spasticity in hemiparetic patients: Grasp strategies and kinematics of reach-to-grasp movements. *Neurorehabilitation and Neural Repair*; 2009; XX(X): I–II. 10.1177/1545968309347683 19786722

[33] Lyle RC. A performance test for assessment of upper limb function in physical rehabilitation treatment and research. *Int J Rehabil Res*; 1981; 4:483–92. 7333761 10.1097/00004356-198112000-00001

[34] Platz T, Pinkowski C, van Wijck F, Kim IH, di Bella P, Johnson G. Reliability and validity of arm function assessment with standardized guidelines for the Fugl-Meyer test, Action Research Arm Test and Box and Block Test: a multicentre study. *Clin Rehabil*; 2005; 19(4):404–11. doi: 10.1191/0269215505cr832oa 15929509 10.1191/0269215505cr832oa

[35] Fugl-Meyer AR, Jääskö L, Leyman I, Olsson S, Steglind S. The post-stroke hemiplegic patient. 1. A method for evaluation of physical performance. *Scand J Rehabil Med*; 1975; 7(1):13–31. 1135616

[36] Gladstone DJ, Danells CJ, Black SE. The fugl-meyer assessment of motor recovery after stroke: a critical review of its measurement properties. *Neurorehabil Neural Repair*; 2002; 16(3);232–40. doi: 10.1177/154596802401105171 12234086 10.1177/154596802401105171

[37] Michaelsen SM, Jacobs S, Roby-Brami A, Levin MF. Compensation for distal impairments of grasping in adults with hemiparesis. *Experimental Brain Researchs*; 2004; 157:162–173. 10.1007/s00221-004-1829-x 14985899

[38] Kim WJ, Kumthornthip W, Oh BM, Yang EJ, Paik NJ. Feasibility of video clip analysis on effect of botulinum toxin-A injection for post-stroke upper limb spasticity. *Toxins*; 2013; 5(5):983–91. doi: 10.3390/toxins5050983 23666198 10.3390/toxins5050983 PMC3709274

[39] Zariffa J, Steeves JD. Computer Vision-Based Classification of Hand Grip Variations in Neurorehabilitation. *IEEE*; 2011. 10.1109/ICORR.2011.5975421 22275622

[40] Enjalbert M, Pelissier J, Codine P, Simon L. Prehension in hemiplegic patients. I. Evolution during rehabilitation. Longuitudinal study of 160 cases. *Elsevier*; 1988; 31: 147–154

[41] Tabachnick BG, Fidell LS. Using Multivariate Statistics. 5th ed., Boston: Pearson; 2007

[42] Hoonhorst MH, Rinske H, Nijland RH, van de Berg JS, Emmelot CH, Kollen BJ, Kwakkel G. How do Fugl-Meyer arm motor scores relate to dexterity according to the Action Research Arm Test at 6 months poststroke? *Archives of Physical Medicine and Rehabilitation*; 2015; 96:1845–9. doi: 10.1016/j.apmr.2015.06.009 26143054 10.1016/j.apmr.2015.06.009

[43] Cesari P, Newell KM. The scaling of human grip configurations. *Journal of Experimental Psychology*; 1999; 25(4):927–35. 10464939 10.1037//0096-1523.25.4.927

[44] Eidenmuller S, Randerath J, Goldenberg G, Li Y, Hermsdorfer J. The impact of unilateral brain damage on anticipatory grip force scaling when lifting everyday objects. *Neuropsychologia*; 2014; 61:222–34. doi: 10.1016/j.neuropsychologia.2014.06.026 24978304 10.1016/j.neuropsychologia.2014.06.026

[45] McDonnell MN, Hillier SL, Ridding MC, Miles TS. Impairments in precision grip correlate with functional measures in adult hemiplegia. *Clinical Neurophysiology*; 2006; 117(7):1474–80. doi: 10.1016/j.clinph.2006.02.027 16679058 10.1016/j.clinph.2006.02.027

[46] Lang CE, Schieber MH. Reduced muscle selectivity during individuated finger movements in humans after damage to the motor cortex or corticospinal tract. *J Neurophysiol*; 2004; 91: 1722–33. doi: 10.1152/jn.00805.2003 14668295 10.1152/jn.00805.2003

[47] Christel MI, Billard A. Comparison between macaques' and humans' kinematics of prehension: the role of morphological differences and control mechanisms. *Behavioural Brain Research*; 2002; 131(1–2):169–84. 11844584 10.1016/s0166-4328(01)00372-2

[48] Marske MW, Marszke RF. Evolution of the human hand: approach to acquiring, analysing, and interpreting the anatomical evidence. *Journal of Anatomy*; 2000; 197:121–140. doi: 10.1046/j.1469-7580.2000.19710121.x 10999274 10.1046/j.1469-7580.2000.19710121.x PMC1468111

[49] Maier MA, Armand J, Kirkwood PA, Yang HW, Davis JN, Lemon RN. Differences in the corticospinal projection from primary motor cortex and supplementary motor area to macaque upper limb motoneurons: an anatomical and electrophysiological study. *Cerebral Cortex*; 2002; 12(3):281–96. 11839602 10.1093/cercor/12.3.281

[50] Lemon RN, Kirkwood PA, Maier MA, Nakajima K, Nathan P. Direct and indirect pathways for corticospinal control of upper limb motoneurons in the primate. *Progress in Brain Research*; 2004; 143:263–79. doi: 10.1016/S0079-6123(03)43026-4 14653171 10.1016/S0079-6123(03)43026-4

[51] Maier MA, Shupe LE, Fetz EE. Dynamic neural network models of the premotoneuronal circuitry controlling wrist movements in primates. *Journal of Computational Neuroscience*; 2005; 19(2):125–46. doi: 10.1007/s10827-005-0899-5 16133816 10.1007/s10827-005-0899-5

[52] York GK, Steinberg DA. Hughlings Jackson’s theory of recovery. *Neurology*; 45:834–838, 1995 7723983 10.1212/wnl.45.4.834

[53] Wiesendanger M, Serrien DJ. Neurological problems affecting hand dexterity. *Brain Research Reviews*; 2001; 36:161–168. 11690612 10.1016/s0165-0173(01)00091-1

[54] Bernstein NA. On dexterity and its development In: Latash ML.; Turvey MT., editors. Dexterity and Its Development. Erlbaum Publ; Mahwah, NJ; 1996; p. 1–244.

[55] Bonaiuto J, Arbib MA. Learning to grasp and extract affordances: the integrated learning of grasp and affordances (ILGA) model. *Biological Cybernetics*; 2015; 109(6):639–69. doi: 10.1007/s00422-015-0666-2 26585965 10.1007/s00422-015-0666-2 PMC4656720

[56] Cesari P, Newell KM. Scaling the components of prehension. *Motor Control*; 2002; 6(4):347–65. 12429890 10.1123/mcj.6.4.347

[57] Gibson AR, Horn KM, Pong M, Van Kan PL. Construction of a reach-to-grasp. *Novartis Foundation symposium*; 1998; 218:233–45. 9949824 10.1002/9780470515563.ch13

[58] Jeannerod M. Specialized channels for cognitive responses. *Cognition*; 1981; 135–137. 7198527 10.1016/0010-0277(81)90036-6

[59] Wilson JN. Providing automatic grasp by flexor tenodesis. *Journal of Bone and Joint Surgery*; 1956; 38-A(5):1019–24. 13367079

[60] Wagner JM, Lang CE, Sahrmann SA, Hu Q, Bastian AJ, Dromerick AW. Relationships between sensorimotor impairments and reaching deficits in acute hemiparesis. *Neurorehabilitation and Neural Repair*; 2006; 20:406–416. doi: 10.1177/1545968306286957 16885427 10.1177/1545968306286957

[61] Bowden JL, Gaven GL, McNulty PA. The prevalence and magnitude of impaired cutaneous sensation across the hang in the chronic period post-stroke. *PLoS ONE*; 2014; 9(8):e104153 doi: 10.1371/journal.pone.0104153 25121607 10.1371/journal.pone.0104153 PMC4133225

[62] Hill VA, Fisher T, Schmid AA, Crabtree J, Page SJ. Relatitonship between touch sensation of the affected hand and performance of valued activities in individuals with chronic stroke. *Topics Stroke Rehabilitation*; 2014; 21(4):339–346. 10.1310/tsr2104-339 25150666

[63] Semrau JA, Herter TM, Scott SH, Dukelow SP. Examining Differences in Patterns of Sensory and Motor Recovery After Stroke With Robotics. *Stroke*; 2015; 46(12):3459–69. doi: 10.1161/STROKEAHA.115.010750 26542695 10.1161/STROKEAHA.115.010750

[64] Ada L, O’Dwyer N, O’Neil E. Relation between spasticity, weakness and contracture of the elbow flexors and upper limb activity after stroke: an observational study. *Disability and Rehabilitation*; 2006; 28(13–14):891–7. doi: 10.1080/09638280500535165 16777777 10.1080/09638280500535165

[65] Leonard CT, Gardipee KA, Koontz JR, Anderson JH, Wilkins SA. Correlation between impairment and motor performance during reaching tasks in subjects with spastic hemiparesis. *Journal of Rehabilitation medicine*; 2006; 38(4):243–9. doi: 10.1080/16501970600609808 16801207 10.1080/16501970600609808

[66] Shaw LC, Price CI, van Wijck FM, Shackley P, Steen N, Rodgers H et al Botulinum toxin for the upper limb after stroke (BoTULS) trial: effect on impairment, activity limitation, and pain. *Stroke*; 2011; 42(5):1371–9. doi: 10.1161/STROKEAHA.110.582197 21415398 10.1161/STROKEAHA.110.582197

[67] Roby-Brami A, Feydy A, Combeaud M, Biryukova EV, Bussel B, Levin MF. Motor compensation and recovery for reaching in stroke patients. *Acta Neurologica Scandinavica*; 2003; 107(5):369–81. 12713530 10.1034/j.1600-0404.2003.00021.x

