# Supplementary material for: Functional classification of grasp strategies used by hemiplegic patients
Source: PLoS One. 2017 Nov 10;12(11):e0187608. doi: 10.1371/journal.pone.0187608 (PMC5695285; doi:10.1371/journal.pone.0187608)
Supplement: S1 Text — (PDF) [file pone.0187608.s001.pdf]

Functional tests used in clinical rehabilitation.

Fugl-Meyer test (FM).

The Fugl-Meyer upper extremity test was derived from the natural history of motor recovery following stroke. After a stroke induced lesion of the motor areas of the brain, paralysis occurs in the body on the opposite side to the lesion (hemiplegia). During the weeks following the stroke, the paralysis usually recovers spontaneously, but partially and inconstantly, and many patients are left with impaired movement (hemiparesis). The symptoms are motor weakness and loss of individual joint control, associated with positive symptoms such as spasticity, co-contraction and pathological synergies. In the early stages of recovery, global and stereotyped patterns of movement emerge, typically shoulder elevation coupled with elbow flexion (*flexor synergy*) or shoulder adduction/internal rotation coupled with elbow extension (*extensor synergy*). If recovery continues, patients become able to execute more precise and focused voluntary movements. Accordingly, during the FMA-UE test, the examiner asks the patient to execute volitional movements, and the capacity to perform the movement with our without the use of synergies or compensations is rated from 0 to 2). Nine items correspond to synergy-related movements of the shoulder and elbow (i.e. move the hand from the contralateral knee to the ipsilateral ear) and six items to shoulder-elbow movements that mix synergies or are out of synergies (i.e. bring the hand to lumbar spine). Five more items examine wrist movements and 7 evaluate the hand, including the ability to grasp objects. The quality of motion during these tasks is also examined by three items (speed, precision and smoothness) and reflex activity and spasticity are rated in 3 items. Thus the section of the upper extremity section of the FMA consists of 33 items, giving a maximum score of 66. The FMA test is valid and reliable when administered by trained therapist (Platz, Pinkowski, Kim, di Bella, & Johnson, 2005), it is widely used in clinical practice to assess the severity of the impairment, to document recovery and to plan and evaluate therapy.

More details on the FMA-UE test can be found in https://www.strokengine.ca/assess/fma/ and its full description on http://neurophys.gu.se/digitalAssets/1520/1520603_fma-ue-protocol-english-updated-20150311.pdf

Action Research Arm Test (ARAT).

In contrast to the FMA-UE test, the ARAT is based on functional tasks involving object manipulation administered in a standardized environment (table, shelf, chair, set of objects). It consists of 19 items grouped into four subcategories: grasp, grip, pinch, and gross movement. The items in each subcategory are ordered according to ascending difficulty. The grasp tasks (6 items) involve grasping and displacing objects to a shelf 37 cm above the table (wooden blocks of 2.5, 5, 7.5 and 10cm^3, a cricket ball and a sharpening stone). The grip subcategory (4 items) involves pouring water from one glass to another, displacing alloy tubes (2.25 and 1 cm diameters 11.5 cm long) fitted on a peg to another peg placed at a distance of 37 cm, and turning a washer on a bolt. The pinch subscale (6 items) consist of grasping a marble (1.5 cm or 0.6 diameter) using two fingers (the thumb and the second, third or fourth fingers) and placing it in a receiver on the shelf. The gross movement subcategory (3 items) consists of placing the hand behind the head, on the top of the head or on the mouth. Each item is scored from 0 to 3 (0 = unable to perform, 1 = performs partially, 2 = completes but abnormal posture or abnormally long, time, 3 = performs the test normally) so that the maximum score is 57.

The ARAT test is also valid and reproducible (Platz, Pinkowski, Kim, di Bella, & Johnson, 2005), Yozbatiran et al. 2008) when administered in a standardized way by trained therapists.

Yozbatiran N, Der-Yeghiaian L, Cramer SC. A standardized approach to performing the action research arm test. Neurorehabil Neural Repair. 2008 Jan-Feb;22(1):78-90. Epub 2007 Aug 17.

More details on the ARAT test can be found in https://www.strokengine.ca/assess/arat/
