# Supplementary figures and images for: Functional classification of grasp strategies used by hemiplegic patients
Source: PLoS One. 2017 Nov 10;12(11):e0187608. doi: 10.1371/journal.pone.0187608 (PMC5695285; doi:10.1371/journal.pone.0187608)

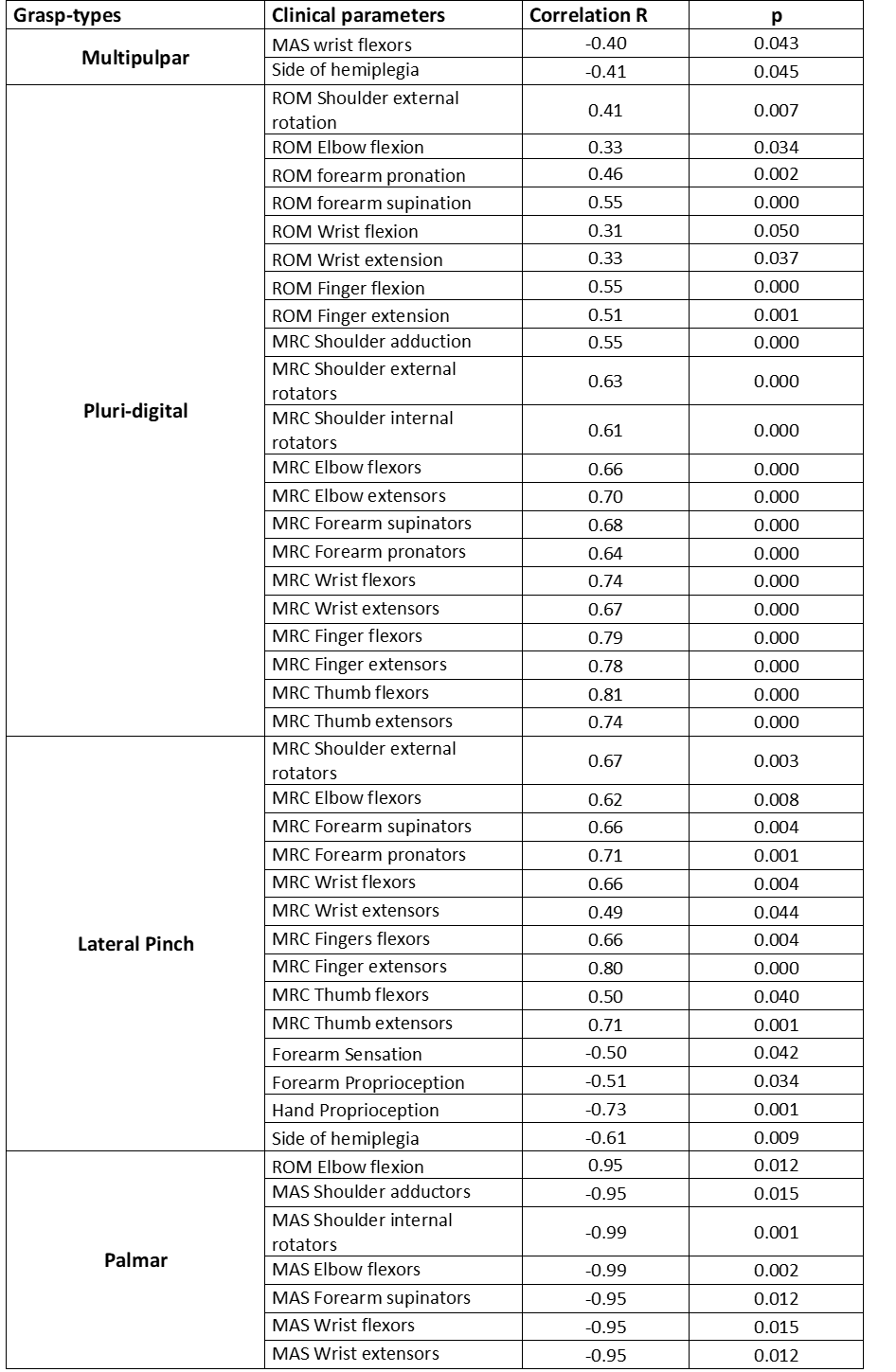

Supplement: S3 Table — (TIF) [file pone.0187608.s004.tif]
